# Health Care Access Barriers Among Reproductive-Age Women in East Africa: Development and Validation of Machine Learning Prediction Models Using DHS Data

**DOI:** 10.2196/85695

**Published:** 2026-09-28

**Authors:** Jenberu Mekurianew Kelkay, Andualem Yalew Aschalew, Getachew Teshale, Melak Jejaw, Kaleb Assegid Demissie, Azmeraw Tadele, Misganaw Guadie Tiruneh, Tesfahun Zemene Tafere, Asebe Hagos, Nebebe Demis Baykemagn

**Affiliations:** 1 Department of Public Health, College of Health Sciences, Debark University, Gondar, Debark, 6000, Ethiopia, 251 0947088025; 2 Department of Health Systems and Policy, Institute of Public Health, College of Medicine and Health Sciences, University of Gondar, Gondar, Ethiopia; 3 Department of Medical Nursing, School of Nursing, College of Medicine and Health Sciences, University of Gondar, Gondar, Ethiopia; 4 Department of Health Informatics, Institute of Public Health, College of Medicine and Health Sciences, University of Gondar, Gondar, Ethiopia

**Keywords:** barriers to health care access, Extreme Gradient Boosting, XGBoost, machine learning, predictive modeling, reproductive-age women, East Africa

## Abstract

**Background:**

The World Health Organization advises that every nation should take responsibility for guaranteeing access to health care services as a basic human right. However, due to financial constraints and geographical hurdles, only around half of the population in Africa has access to contemporary health care services.

**Objective:**

This study aimed to predict barriers to health services and associated factors among reproductive-aged women in East Africa using machine learning algorithms and identify the best-performing predictive model.

**Methods:**

Analysis of secondary data from 6 East African countries using the Demographic and Health Surveys from 2016 to the recent 2023 was performed. A weighted total sample of 228,654 women of reproductive age was included in this study. Data were extracted and processed with Stata version 17. The dataset was then imported into a Jupyter notebook for further detailed analysis and visualization. A machine learning algorithm using different classification models was implemented. All analyses and calculations were performed in the Python 3 programming language in Jupyter Notebook using imblearn, scikit-learn, and Extreme Gradient Boosting (XGBoost) packages.

**Results:**

Among 228,654 reproductive-age women included in the study, the XGBoost classifier demonstrated the best predictive performance, with 94.46% accuracy, 94.62% precision, 93.73% recall, 94.17% *F*_1_-score, and 98% area under the curve. Overall, 92.63% (211,794/228,654) of women experienced barriers to health care access. Predictors of barriers to health access were identified using an XGBoost model with the help of Shapley Additive Explanations. This study showed that maternal occupation, maternal age, low media exposure, parity, marital status, health insurance, and community literacy were the top predicting factors of barriers to health access.

**Conclusions:**

The XGBoost model demonstrated the best predictive performance among the evaluated algorithms. The findings indicate that a substantial proportion of reproductive-age women experience barriers to health care access, although no formal subnational “extreme risk” classification was conducted in this study. Enhancing comprehensive health education and reducing financial barriers through the expansion of health insurance coverage may help improve health care access, particularly among vulnerable populations such as rural women.

## Introduction

Access to health care services is a basic human right, and obstacles to obtaining these services can negatively impact an individual’s physical and mental well-being, as well as their overall quality of life [1]. Individuals’ health, along with their physical, emotional, and social well-being, is influenced by their availability of health care [2]. Unfortunately, access to health care services remains a widespread challenge in low- and middle-income countries [3].

Globally, an estimated 400 million people lack access to essential health care services, and approximately 8 million deaths each year are due to conditions that are preventable or treatable with timely and adequate medical care [4]. Due mainly to financial constraints and geographical hurdles, only around half of the population in Africa has access to contemporary health care services [5]. Women have limited access to health care due to many issues, including the need for spousal consent, financial constraints, and the distance to medical facilities [4].

Less than half of mothers in East Africa receive support from trained birth attendants, and the region’s overall maternal health metrics continue to perform poorly [6]. Reducing maternal death and morbidity requires increasing access to and use of basic health care services. However, maternal health services coverage is low in sub-Saharan Africa (SSA) due to women’s barriers to health care access and use of essential health services. The health and survival of mothers and their infants are adversely affected by inadequate use of the vital continuum of care [7]. In Ethiopia, significant barriers to health care access persist, especially in rural communities. In order to address these issues, the government has increased access to primary health care services by building health centers and health posts quickly and by deploying and educating primary and midlevel health care workers [8].

To enhance maternal health outcomes, several regional and international efforts have been implemented, including the Sustainable Development Goals and the Millennium Development Goals [9]. Despite significant efforts made both internationally and at the local level, the health status of mothers has not improved to the anticipated extent. However, disparities in regions and socioeconomic discrepancies continue to exist in health care access, particularly for those living in rural areas. Health care accessibility is known to be influenced by a wide range of factors, such as patient acceptance of treatments [10], physician preferences [11], place of residence [12], religious beliefs, and the empowerment of women [13].

The European Commission (2014) Communication states that several factors, such as population coverage, affordability, the variety of services provided, and the accessibility of care (such as waiting times and distance), characterize access to health services [14]. These elements are deeply interconnected. Meanwhile, the Demographic and Health Survey (DHS) statistics guide assesses reproductive-age women’s access to health care by posing several questions about the main obstacles they might encounter when seeking medical attention while ill, including financial hardship, obtaining authorization to seek treatment, the distance to medical facilities, and the capacity to travel alone [15].

Previous studies examining barriers to health care access have primarily relied on conventional statistical approaches such as logistic regression and multilevel modeling. Although these methods are valuable for identifying associations between variables, they often depend on predefined assumptions, linear relationships, and hypothesis-driven model structures. Such approaches may have limited capacity to detect complex nonlinear interactions, hidden patterns, and high-dimensional relationships within large population datasets. Therefore, this study used multiple machine learning (ML) algorithms to predict barriers to health care access and identify the most effective predictive model [16]. Various classification techniques were evaluated and compared, and Shapley Additive Explanations (SHAP)–based explainability was used to improve model interpretability and identify the most influential predictors [17]. ML has dominated many fields in recent years due to its capacity to identify hidden patterns in data, enabling the constructive extrapolation of such patterns to previously unobserved fresh data [18,19].

Several ML classifiers, including logistic regression, random forest, decision tree, k-nearest neighbor (KNN), support vector machine (SVM), artificial neural network (ANN), categorical boosting (CatBoost), Light Gradient Boosting Machine (LightGBM), Extreme Gradient Boosting (XGBoost; XGBoost developers, open-source software), and a stacking ensemble model, were evaluated [20]. Comparative model assessment was conducted using cross-validation and independent test-set performance metrics. A key innovation of this approach is the integration of SHAP, which enhances model interpretability by quantifying the contribution of individual predictors to model outputs. This interpretable ML framework provides transparent insights into the key determinants of health care access barriers while maintaining strong predictive performance.

## Methods

### Data Source and Study Population

The DHS used a population-based cross-sectional survey design to collect data, and this study used a predictive modeling approach. Secondary data from DHS datasets from 2016 to the recent 2023 were considered for this analysis. This study followed Transparent Reporting of a Multivariable Prediction Model for Individual Prognosis or Diagnosis-AI reporting guidelines for prediction model development and reporting [21]. Eastern Africa is the eastern subregion of the African continent in the United Nations Statistics Division scheme of geographic regions. Six East African countries, namely Burundi, Ethiopia, Madagascar, Uganda, Rwanda, and Zambia, were included in this study.

Following authorization through an online request that explains the goal of our study, the data can be accessed from the DHS program’s official database [22]. All of the included factors were derived from the dataset of women’s individual records. Every 5 years, a nationally representative DHS data collection is carried out in low- and middle-income countries [23]. Maternal health-related reproductive concerns are the primary focus of the data. In essence, a stratified sampling procedure with 2 stages was used. For the analysis, a total of 228,654 women of reproductive age were weighted.

### Ethical Considerations

Since the study was a secondary data analysis, participant consent was not necessary. Following submission of the consent paper to the DHS program, the International Review Board of the DHS program data archivists waived informed consent, and a letter of permission was granted to download the dataset for this study. The International Review Board–approved procedures for DHS public-use datasets do not allow respondents, households, or sample communities to be identified. There are no names of individuals or household addresses in the data files.

### Study Variables

#### Dependent Variable

In this study, the dependent variable was barriers to health care access. Four questions that addressed the difficulties women could face when seeking medical attention were used to measure this variable. These difficulties included an inability to pay for treatment, a long commute to a medical center, a lack of authorization to seek care, and a lack of a companion. Women were classified as experiencing barriers to health care access if they reported at least one of these issues. Individuals who reported none of these issues were coded as 0 and deemed to have no barriers [24].

#### Independent Variables

Various independent variables were examined in this study to identify factors associated with barriers to health care access. These included health insurance, respondent age, respondent education, marital status, wealth status, parity, husband education, respondent occupation, husband occupation, community literacy, residency, household head, and media exposure (Table 1).

**Table 1.** List of variables for the assessment of barriers to health care access among reproductive-age women in East Africa.

Variable	Categories and definitions
Place of residence	Urban and rural
Mother’s education	No formal education, primary education, secondary, and above
Mother’s age (y)	15‐24, 25‐34, and 35‐49
Husband’s education	No formal education, primary education, secondary, and above
Mother’s occupation	Not working and working
Sex of household head	Male and female
Marital status	Married and not married
Community literacy	Low, medium, and high. Derived by aggregating women’s education levels in each cluster
Health insurance	Yes and no
Media exposure	Exposed (access to newspaper, radio, or TV) and unexposed
Attitude toward wife beating	Accept (justifies in at least one condition) and reject
Distance to health facility	Big problem and not a big problem (based on respondent’s perception)
Parity (children)	No parity (0), multiparity (1‐4), and grand multiparity (5 or more)

#### Data Management and Analysis

Stata (version 17; StataCorp LLC) was used for the initial data extraction, while Jupyter Notebook was used for further processing. Sampling weights were retained from the original DHS observations and incorporated into model training using the sample_weight parameter after applying Synthetic Minority Oversampling Technique (SMOTE)–Tomek only to the training data within each fold. To preserve data integrity, the dataset was thoroughly cleaned, including missing entry imputation. In order to avoid skewing the results, extreme values were identified and eliminated. Python 3 was used for all further calculations and modeling in Jupyter Notebook, using the imblearn [25], scikit-learn (scikit-learn developers, open-source software) [26], XGBoost [27], and SHAP [28] packages. All models and analytic procedures were executed in Jupyter Notebook using Python 3.10.11. Because this study used predictive ML methods rather than inferential statistical modeling, traditional hypothesis testing and *P* values were not applicable and therefore were not reported.

To prepare the data for modeling, preprocessing included feature selection to reduce noise and redundancy, and standard scaling to normalize feature contributions. Class imbalance was addressed using SMOTE-Tomek, applied strictly within the training folds to prevent data leakage, together with class weighting to further handle minority classes. All preprocessing steps were implemented within a unified pipeline to ensure transformations were learned only from the training data, preserving the validity of model evaluation [29].

The dataset was then split into training and testing sets using an 80:20 ratio to facilitate model training and evaluation. To further ensure the robustness of the model, k-fold cross-validation was used, allowing the dataset to be divided into k subsets [30]. In each iteration, a different subset served as the validation set, while the remaining subsets were used for training. Group-aware cross-validation was performed using primary sampling units as grouping variables to ensure that observations from the same cluster were not shared between training and test folds, thereby reducing potential information leakage due to intracluster correlation. The evaluation metrics obtained from each fold were averaged to assess the model’s generalization performance. This validation process helps detect overfitting and ensures the model’s reliability when applied to unseen data [31].

### Data Splitting and Resampling

#### Training-Test Split and SMOTE-Tomek Resampling

The dataset was first divided into training (182,923/228,654, 80%) and testing (45,731/228,654, 20%) subsets before any resampling procedures were performed. The independent test dataset remained completely untouched and was used only for final model evaluation. To address class imbalance, SMOTE-Tomek resampling was applied exclusively to the training data within each cross-validation fold. Synthetic observations generated during resampling were used solely for model training and were not assigned DHS survey weights because they did not represent actual survey respondents. Survey weights were retained only for analyses requiring population-representative estimates.

#### ML Model Development and Comparison

ML models were developed and compared to identify the most accurate classifier for predicting barriers to health care access. The evaluated algorithms included logistic regression, random forest, decision tree, KNN, SVM, ANN, CatBoost, LightGBM, XGBoost, and a stacking ensemble model. Model performance was assessed using accuracy, precision, recall, *F*_1_-score, and area under the receiver operating characteristic curve (AUC-ROC). The stacking model was included as a benchmark ensemble approach, whereas XGBoost was selected as the final predictive model because it demonstrated the highest overall performance on the independent test dataset (Figure 1).

**Figure 1.**
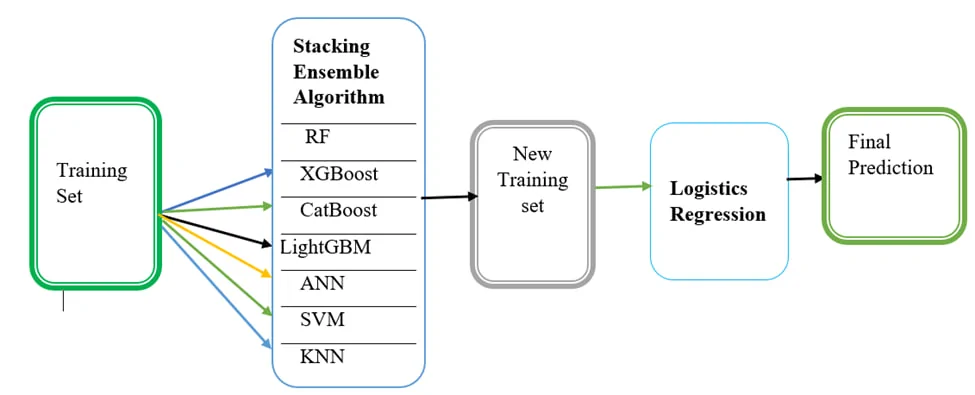
Machine algorithm workflow. ANN: artificial neural network; CatBoost: categorical boosting; KNN: k-nearest neighbor; LightGBM: Light Gradient Boosting Machine; RF: random forest; SVM: support vector machine; XGBoost: Extreme Gradient Boosting.

#### Evaluation Metrics

The dataset used for this analysis falls under binary classification, as the target variable, barriers to health access, was divided into 2 mutually exclusive categories. To build predictive models, different classification algorithms were applied: logistic regression, random forest, KNN, LightGBM, ANN, SVM, CatBoost, XGBoost, and decision tree [32]. About country-specific performance trends, these algorithms were chosen for their efficacy in previous research that used ML techniques on DHS data for categorization tasks (Table 2).

**Table 2.** Confusion matrix and multiple derived metrics adapted from [33].

Actual	Predictive positive	Predictive negative
Positive	True positive (TP)	False negative (FN)
Negative	False positive (FP)	True negative (TN)

Based on the confusion matrix above, the following metrics were derived: sensitivity (recall), specificity, accuracy, and precision.


Recall(sensitivity)=TP/(TP+FN)

Specificity=TN/(TN+FP)

Precision=TP/(TP+FP)

$$F_{1}-score=2\times \left(Precision\times Recall\right)/\left(Precision+Recall\right)$$

Accuracy=(TP+TN)/(TP+TN+FP+FN)

Recall ensures that the majority of minority class instances are correctly identified, minimizing false negatives (FNs). However, the tradeoff is that a high-recall model may have lower precision, resulting in more false positives (FPs). Precision reflects the proportion of predicted positives that are truly correct. High precision is crucial when FPs carry serious consequences. The tradeoff is that maximizing precision can reduce recall, as the model becomes more cautious in labeling positives. The *F*_1_-score balances precision and recall by calculating their harmonic mean, making it useful when both FPs and FNs are equally important [34]. However, the tradeoff is that the *F*_1_-score can obscure imbalances if one metric is much higher than the other. Accuracy measures overall prediction correctness but can be deceptive in imbalanced datasets. The tradeoff is that accuracy does not distinguish between errors in the majority and minority classes.

To evaluate predictive performance, we used a confusion matrix to analyze classification results through true positives (TPs), FPs, and FNs. To improve the interpretability of the final predictive model, SHAP were applied to the XGBoost classifier. SHAP values were used to quantify the contribution of each predictor to model predictions and to identify the most influential factors associated with health care access barriers [35]. Additionally, model performance was compared using the AUC-ROC, which graphically represents the trade-off between TP rates and FPs across classification thresholds. The confusion matrix implementation followed established methodology [36], while SHAP and AUC-ROC provided complementary insights into feature contributions and overall classification performance.

Recall reduces FNs by ensuring that the majority of cases in the minority class are identified. A model with high recall may trade off precision for a higher number of FPs. Precision is the percentage of predicted positives that turn out to be accurate. When FPs have significant effects, high precision is crucial. Tradeoff: because the model becomes more cautious when making positive predictions, maximizing precision may result in a decrease in recall. The *F*_1_-score balances the trade-off between precision and recall by taking the harmonic mean of the two. Useful when both FPs and FNs are equally critical. Tradeoff: the *F*_1_-score can mask imbalances if one metric is significantly higher than the other. Accuracy measures the overall correctness of predictions but can be misleading in imbalanced datasets. Tradeoff: accuracy does not differentiate between errors in the majority and minority classes.

### Association Rule Mining

Using association rule mining (ARM), nontrivial and perhaps hidden patterns among the independent variables linked to health care access barriers for women of reproductive age were found [37]. Instead of focusing on the direction and strength of individual predictors as typical regression models do, ARM finds combinations of characteristics (rules) that often occur together and are highly related to a particular outcome, in this case, reported barriers to health care access. The Apriori algorithm was used to apply the ARM approach, with support, confidence, and lift levels predetermined. A rule’s strength over chance is indicated by lift, its frequency in the dataset is measured by support, and its probability of occurring given the antecedent is reflected by confidence.

## Results

### Sociodemographic Characteristics

A total of 228,654 reproductive-age women were included in the study. Of these, 162,872 (71.23%) were from rural areas. Nearly half of the participants, 94,980 (41.54%), were aged 15 to 24 years. Regarding educational status, 100,924 (44.14%) women had primary education, whereas 62,173 (27.19%) had no formal education (Table 3).

**Table 3.** Sociodemographic characteristics of barriers to health care access among women in East Africa (N=228,654).

Index and variable	Weighted frequency, n (%)
1. Place of residence	
Urban	65,782 (28.77)
Rural	162,872 (71.23)
2. Mother’s education	
No formal education	55,890 (24.44)
Primary education	100,924 (44.14)
Secondary and above	71,840 (31.42)
3. Mother’s age (years)	
15‐24	94,980 (41.54)
25‐34	69,542 (30.41)
35‐49	64,132 (28.05)
4. Husband’s education^a^	
No formal education	38,958 (17.04)
Primary education	101,845 (44.54)
Secondary and above	72,345 (31.64)
5. Mother’s occupation	
Not working	73,938 (32.34)
Working	154,716 (67.66)
6. Sex of household head	
Male	161,616 (70.68)
Female	67,038 (29.32)
7. Marital status	
Married	108,350 (47.39)
Not married	120,304 (52.61)
8. Community literacy	
Low	107,266 (46.91)
Medium	1616 (0.71)
High	119,772 (52.38)
9. Health insurance	
No	201,566 (88.15)
Yes	27,088 (11.85)
10. Media exposure	
Unexposed	87,142 (38.11)
Exposed	141,512 (61.73)
11. Wife beating	
Accept	74,644 (32.64)
Reject	154,010 (67.36)
12. Wealth status	
Poor	87,346 (38.20)
Middle	39,196 (17.14)
Rich	102,112 (44.66)

a The data on husband’s education was available for 213,148 respondents; 15,506 respondents had missing or unavailable information and were therefore excluded from this variable.

### Socioeconomic Characteristics

According to the participants’ wealth index distribution, nearly half (102,112/228,654, 44.66%) were categorized as high-income, 38.20% (87,346/228,654) as low-income, and only 17.14% (39,196/228,654) as middle-income. The large percentage of participants with low and middle incomes draws attention to possible obstacles in obtaining health care and other essential services due to financial limitations. Regarding media exposure, 141,152 out of 228,654 (61.73%) participants were exposed to media sources such as television, radio, or newspapers, whereas 87,142 (38.11%) participants had no media exposure (Table 3).

### ML Analysis of the Barrier to Health Access

Multiple feature selection methods were applied, including Boruta, recursive feature elimination, mutual information, and variance threshold, to evaluate their impact on model performance. A baseline model was used for comparison. To avoid data leakage, feature selection was performed within a nested cross-validation framework, where selection was applied only to the training folds and evaluated on the corresponding test folds. The exact feature lists are provided in Figure S1 in Multimedia Appendix 1. Substantial overlap was observed among the methods, indicating consistent identification of key predictors.

### Health-Related Service Characteristics

Of the 228,654 total sample, only 27,088 (11.85%) mothers had health insurance coverage. About 53,730 (23.50%) of women have a big problem with the distance to health facilities (Table 4). Around 93% (n=211,802) of reproductive-age women reported at least 1 barrier in East Africa.

**Table 4.** Health-related service characteristics of women in East Africa (N=228,654).

Index and variable	Weighted frequency, n (%)
1. Health Insurance	
Yes	27,088 (11.85)
No	201,566 (88.15)
2. Distance to health facility	
Big problem	53,730 (23.50)
Not big problem	174,924 (76.50)
3. Parity	
No	70,252 (30.72)
Multi	104,452 (45.68)
Grand multiparity	53,950 (23.59)

### Balancing and Unbalancing

Handling class imbalance was an essential step in this study to improve the predictive performance of ML algorithms. The survey-weighted DHS dataset consisted of 228,654 observations, of which 211,794 (92.63%) were classified as experiencing at least one barrier to health care access and 16,860 (7.37%) were classified as having no barriers before any data-balancing procedures. This substantial class imbalance had the potential to bias model predictions toward the majority class.

To address this issue, the dataset was first divided into training (182,923/228,654, 80.0%) and testing (45,731/228,654, 20.0%) subsets. The independent test set remained untouched throughout model development and evaluation. To mitigate class imbalance during model training, resampling techniques, including undersampling of the majority class and oversampling of the minority class, were applied as appropriate. Additionally, the SMOTE was applied only to the training dataset to generate synthetic minority-class observations. This process improved class balance in the training data, reduced model bias toward the majority class, and enhanced classification performance while preventing information leakage (Figure 2).

**Figure 2.**
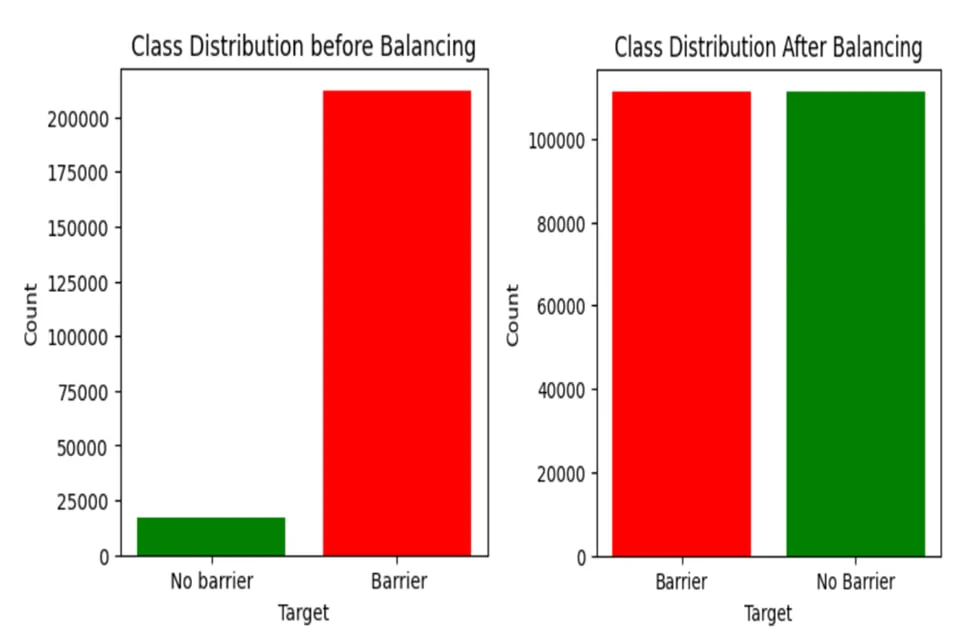
Resampling target variable barrier to health care access.

### Model Development and Evaluation

After the data was cleaned and balanced, we divided it into 2 sets: 80.0% (182,923/228,654) for training and 20.0% (45,731/228,654) for testing. In order to forecast barriers to health access, we developed and compared multiple ML classification models. Among the evaluated algorithms, XGBoost demonstrated the highest predictive performance and was selected as the final model. We balanced each model and then examined each to determine which one performed the best. Table 5 demonstrates that predictive performance was much enhanced by balancing the target variable. With an accuracy of 94.46%, precision of 94.62%, recall of 93.73%, and *F*_1_-score of 94.17%, the XGBoost model was superior when using the SMOTE approach.

**Table 5.** Model performance comparison.

Algorithm	Accuracy (%)	Precision (%)	Recall (%)	*F*_1_-score (%)
XGBoost^a^	94.46	94.62	93.73	94.17
Random forest	94.10	94.52	93.63	94.13
Logistic model	88.41	88.44	88.37	88.41
LightGBM^b^	40.00	27.78	50.00	35.71
CatBoost^c^	93.81	94.52	93.03	93.77
ANN^d^	85.00	82.00	88.00	85.00
SVC^e^	36.67	23.53	40.00	29.63
KNN^f^	90.72	92.05	89.13	90.57
Decision tree	93.39	95.03	91.57	93.27
Stacking	93.01	94.86	93.71	93.21

a XGBoost: Extreme Gradient Boosting.

b LightGBM: Light Gradient Boosting Machine.

c CatBoost: categorical boosting.

d ANN: artificial neural network.

e SVC: support vector classifier.

f KNN: k-nearest neighbor.

Of the individual base models incorporated into the stacking framework, XGBoost exhibited the highest independent predictive performance. We incorporated XGBoost into a stacking ensemble with other models like random forest, CatBoost, ANN, SVM, and KNN, instead of depending on it alone. The final prediction was generated by a logistic regression meta-model that took the predictions from all base models as inputs. This stacking method used the advantages of XGBoost and enhanced overall performance through the combination of complementary patterns identified by the various learners.

### Visualization of Feature Importance

Traditional statistical approaches use predetermined criteria, such as *P* value thresholds, to identify important variables and adhere to set analytical frameworks. ML models, on the other hand, find patterns in data immediately and dynamically. Although ML algorithms are frequently criticized for being difficult to understand (considered black-box systems), new developments like the SHAP framework [38] offer a methodical approach to explaining their predictions.

In our study, we used SHAP to interpret our ML model and uncover the key drivers of health care access barriers. Feature importance was assessed based on mean SHAP values (Figure 3), where longer horizontal bars represent stronger predictive influence. The results highlighted several critical factors: mother’s occupation, media exposure, maternal age, parity, community literacy, health insurance, and marital status all significantly contributed to the barriers to health access in reproductive-age women.

The ranked relevance of each feature in the predictive model is shown on the vertical axis (y-axis) in Figure 4, and the corresponding SHAP values, a standardized metric measuring the influence of each variable, are shown on the horizontal axis (x-axis). Each feature’s row has colored dots that show the relative contributions of various values to the result: blue denotes low-risk values that lower the predicted risk, while red denotes high-risk values that raise it. The remaining factors had minimal or no impact on incomplete immunization status.

**Figure 3.**
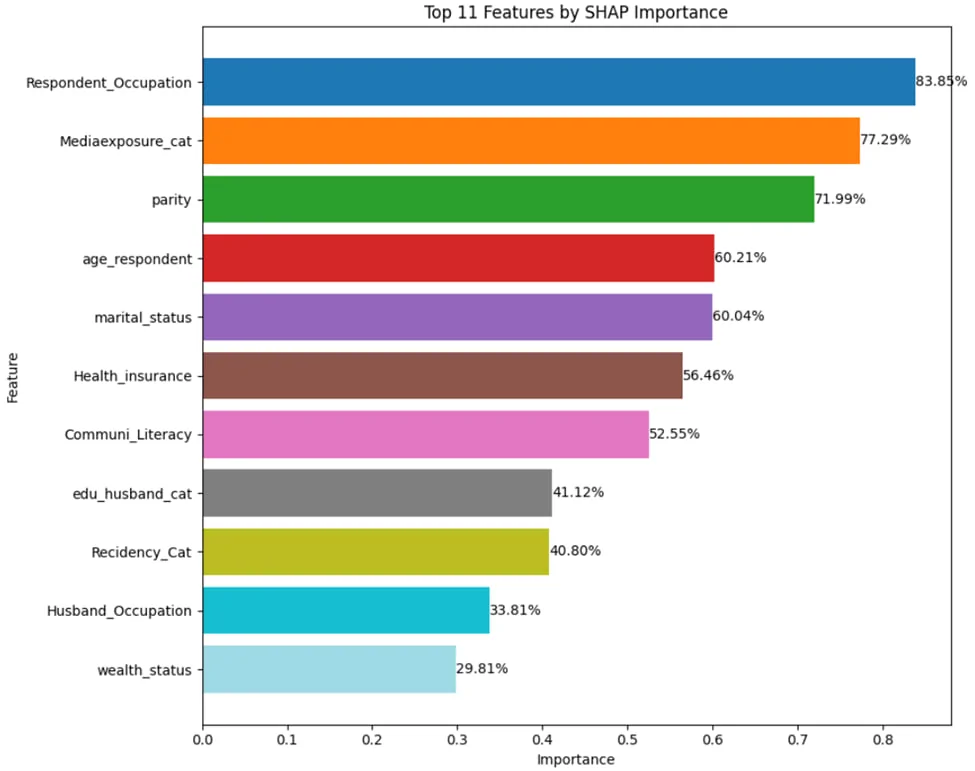
Top 11 features influencing barriers to health care access. SHAP: Shapley Additive Explanations

**Figure 4.**
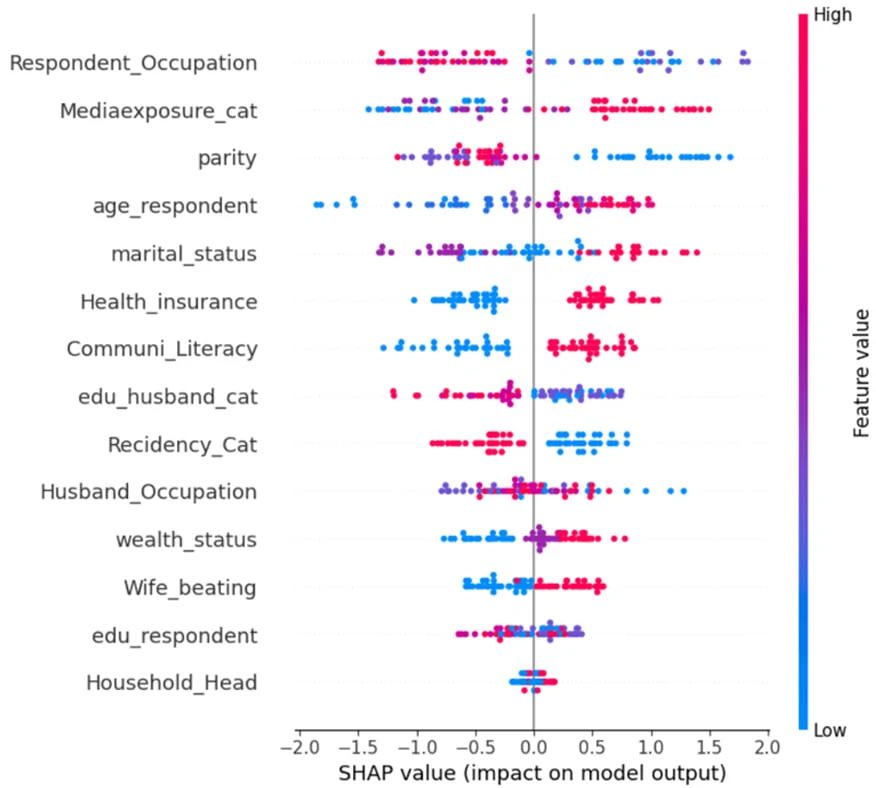
Shapley Additive Explanations (SHAP) summary plot. The x-axis shows SHAP values, where positive values increase the predicted probability of the outcome and negative values decrease it. Each dot represents 1 observation. Color indicates the feature value (blue=low and red=high). Dot dispersion shows the effect direction and magnitude across observations. Features are ranked by the mean absolute SHAP value (top=most influential).

### Predicting Barriers to Health Access

An independent test dataset comprising 45,730 observations was used to evaluate the performance of the XGBoost model. As shown in Figure 5, the model correctly classified 40,509 TPs and 2899 true negatives. In contrast, 1850 cases were FNs and 472 cases were FPs. Overall, the XGBoost model demonstrated strong predictive performance in identifying barriers to health care access among reproductive-age women in East Africa.

The AUC-ROC (Figure 6) was used to evaluate model performance across all thresholds, thereby accounting for varying misclassification error weightings. The XGBoost model, after applying data balancing and hyperparameter optimization, the model’s performance improved significantly, yielding an AUC-ROC of 98% on the test data, indicating strong predictive capability.

**Figure 5.**
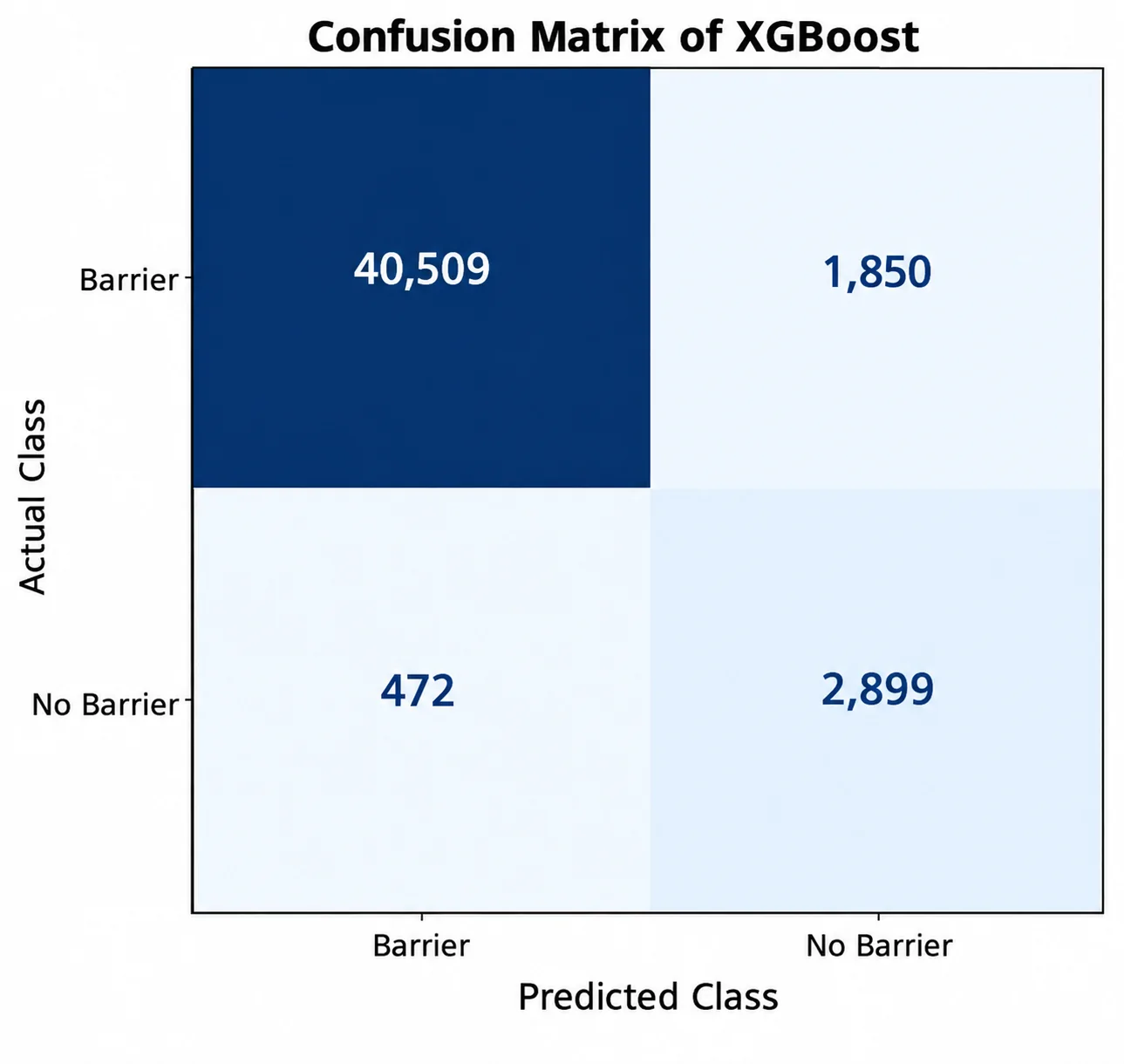
Confusion matrix of the Extreme Gradient Boosting (XGBoost) model prediction on the test data.

**Figure 6.**
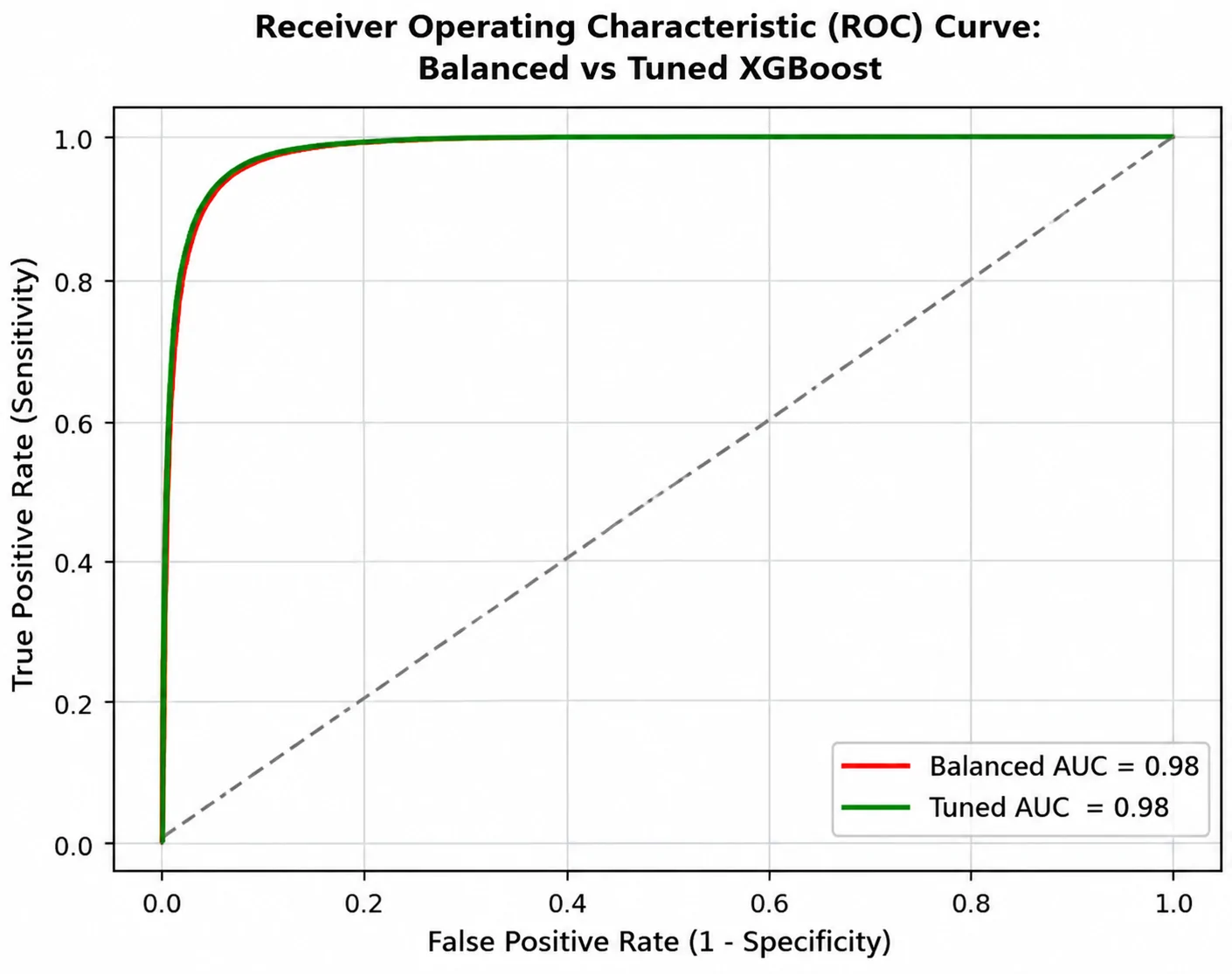
Area under the curve (AUC) of the Extreme Gradient Boosting (XGBoost) model on the test data.

### ARM

ARM was applied as an exploratory technique to identify patterns between socioeconomic and demographic factors and barriers to health care access. The Apriori algorithm was used after discretizing continuous variables into categorical groups, including maternal age (15-24, 25-34, and 35-49) and parity (no parity, multiparity, and grand multiparity).

Minimum support and confidence thresholds were applied to reduce spurious rule generation. The rules, generated from the original (nonresampled) dataset, were evaluated using support, confidence, lift, leverage, and conviction (Table 6). A total of 8 rules were identified, and the top 3 were selected based on support and lift. All selected rules showed 100% confidence, indicating strong subgroup-level associations driven by data discretization. Support values indicate the occurrence within specific subpopulations, while lift values (1.49‐2.10) confirm meaningful associations beyond chance.

**Table 6.** Association rule mining results for the top 3 rules identifying barriers to health care access.

Rule	Antecedent→consequent	Support	Confidence (%)	Lift	Leverage	Conviction
1	No insurance + low literacy→barrier	0.23704	100	1.49	0.08834	1.15742
2	Age 35‐49 y + low literacy→barrier	0.23481	100	2.10	0.12266	2.54427
3	No insurance + age 35‐49 y→barrier	0.06464	100	1.99	0.04873	1.93639

Rule 1 (no health insurance and low community literacy→barrier to health care access) showed support of 0.23704, confidence of 100%, lift of 1.49, leverage of 0.08834, and conviction of 1.15742, indicating a moderate association.

Rule 2 (age 35‐49 y and low community literacy→barrier to health care access) had support of 0.23481, confidence of 100%, lift of 2.10, leverage of 0.12266, and conviction of 2.54427, indicating a strong association.

Rule 3 (no health insurance and age 35‐49 y→barrier to health care access) showed support of 0.06464, confidence of 100%, lift of 1.99, leverage of 0.04873, and conviction of 1.93639, also indicating a meaningful association.

## Discussion

### ML Model Performance and Key Findings

This study was conducted to predict the top risk factors of barriers to health access among reproductive-age women. Multiple ML algorithms were trained on both balanced and imbalanced data for prediction purposes. The performance of those stacking ML models was compared by their classification accuracy and *F*_1_-score. The SMOTE data-balancing technique significantly improved model performance compared to models trained on the original imbalanced dataset, as evidenced by enhancements in accuracy, *F*_1_-score, and AUC-ROC. On the balanced dataset, XGBoost outperformed the other classifiers that were evaluated, predicting with an accuracy of 94.46%, precision of 94.62%, recall of 93.73%, *F*_1_-score of 94.17%, and AUC-ROC of 94%. This suggests a potential limitation of algorithms for this specific dataset. For instance, SVC may struggle with the dataset’s complexity, and LightGBM might require more hyperparameter tuning to match XGBoost’s effectiveness. These differences highlight that tree-based ensemble methods like XGBoost may be better suited for capturing complex patterns in the data [39].

Using SHAP mean values, which measure each feature’s rank of importance in the modified XGBoost model, the most significant characteristics indicating barriers to health access were found. By evaluating each feature’s directional influence on the model’s output, the SHAP impact analysis also demonstrated how each feature contributed to predictions. In the SHAP summary figure, red dots indicated features with a greater predictive probability (which pushed predictions toward a barrier to health access), whereas blue dots at the bottom of the plot indicated features with a lower predictive probability (which were linked to no barrier to health care access). Maternal occupation, maternal age, media exposure, parity, marital status, health insurance, and community literacy were the top predicting factors for barriers to health access among East African reproductive-age women.

Another key objective of this study was to identify specific demographic and socioeconomic categories associated with barriers to health care access among reproductive-age women in East Africa. Using ARM, we analyzed patterns to determine which factors were most strongly linked to restricted health care access. The results revealed that women aged 35 to 49 years, those without health insurance coverage, and those residing in communities with low literacy rates exhibited the highest predictive probability of facing health care access barriers. These findings highlight critical disparities that may require targeted policy interventions to improve health care accessibility for vulnerable groups in the region.

In our study, older mothers (35-49 y) were associated with barriers to health care access among reproductive-age women in East Africa. According to these studies, older women in East Africa encounter major obstacles when trying to secure health care, such as inaccessible services, lack of government assistance, and social or financial difficulties [40]. This finding is consistent with studies conducted in China [41], Ethiopia [42], Nigeria [41], and the United States [41], which reported that older mothers are more likely to face barriers to health care access compared to younger mothers. The possible reason could be that this study was attributed to large samples and included more areas beyond 1 country. This is opposite to a study done in SSA [43]. This might be due to a lack of autonomy in health use, where families may take the role of decision-making to use health care services. Therefore, as women grow older, they tend to gain greater autonomy, financial independence, and decision-making power, which can improve their ability to access health care facilities.

Findings from the rule generation women without health insurance coverage were associated with barriers to health care access in East Africa. A possible explanation could be due to financial limitations, out-of-pocket expenses that are restrictive, especially for women in lower-income circles, which causes care to be postponed or neglected [44], geographic isolation (far facilities and poor transportation) [45], lower education, unemployment, and poverty exacerbate these issues. Additional barriers are brought about by low health insurance awareness, poor-quality services, and cultural conventions [44,45]. These interrelated obstacles necessitate all-encompassing solutions, such as increased insurance coverage, poverty alleviation, improved infrastructure, and health literacy initiatives. This study is similar to what has been reported in several other studies, including in Nigeria [46], Ghana [46], Kenya [46], and East Africa [47].

Our study also shows that communities with lower literacy can be an important factor in the barrier to health care access among reproductive-age women in East Africa. A possible reason could be that limited health literacy in women reduces access to maternal care, antenatal visits, HIV testing, and health insurance [45]. This disadvantage worsens with rural isolation, poverty, and lack of health information, creating overlapping barriers to care [45]. This result is comparable to studies conducted in Tanzania [48] and Ethiopia [4], a systematic review in SSA [49], and Uganda [50].

### Implications of the Study

This study highlights important areas for intervention to increase East African women’s access to health care. Expanded universal health coverage is critical, especially for low-income and rural women, as the strong correlation between lack of insurance and service barriers is evident. In order to reach vulnerable populations, policymakers should give priority to subsidized insurance plans and mobile health services. Furthermore, linking health education with programs for women’s empowerment may improve health care use, as evidenced by the result that community literacy strongly predicts access barriers. Health systems should leverage the study’s high-performing predictive model (XGBoost with 94.46% accuracy) to identify high-risk women early and allocate resources more efficiently.

### Limitations of the Study

DHS data are often cross-sectional, providing a moment in time instead of a long-term perspective. As a result, causal inferences are limited because linkages do not necessarily indicate causation. Data from the DHS may not fully reflect recent changes in patterns or behavior because it collects data at predefined intervals.

### Conclusions

This study developed and evaluated multiple ML algorithms to predict barriers to health care access among reproductive-age women in East Africa. Among the evaluated models, XGBoost demonstrated the best predictive performance and provided interpretable insights into the key determinants of health care access barriers through SHAP analysis. The visualization of cumulative domain-specific feature importance and the graphical representation of influential factors enable policymakers and health service program managers to clearly comprehend the key determinants affecting barriers to health care access rates among reproductive-age women in their respective regions. Before this, maternal occupation, maternal age, media exposure, parity, marital status, health insurance, and community literacy must all be taken into consideration while implementing health policies intended to reduce barriers to health care access. It is essential that all stakeholders, the Africa Union and the Eastern Africa regional coordination center, take appropriate measures to ensure that the health care access process is accessible to all women in the region.

## Supplementary material

10.2196/85695 Multimedia Appendix 1 Comparison of feature selection methods based on the number of selected features and weighted *F*_1_-scores.

## Abbreviations

ANN: artificial neural network
ARM: association rule mining
AUC-ROC: area under the receiver operating characteristic curve
CatBoost: categorical boosting
DHS: Demographic and Health Survey
FN: false negative
FP: false positive
KNN: k-nearest neighbor
LightGBM: Light Gradient Boosting Machine
ML: machine learning
SHAP: Shapley Additive Explanations
SMOTE: Synthetic Minority Oversampling Technique
SSA: sub-Saharan Africa
SVM: support vector machine
TP: true positive
XGBoost: Extreme Gradient Boosting
